# Is SARS-CoV-2 facing constraints in its adaptive evolution?

**DOI:** 10.17305/bb.2025.12537

**Published:** 2025-06-09

**Authors:** Yingguang Liu

**Affiliations:** 1 Department of Biomedical Sciences, College of Osteopathic Medicine, Liberty University, Lynchburg, VA, USA

**Keywords:** Severe acute respiratory syndrome coronavirus 2 (SARS-CoV-2), COVID-19, evolution, mutation, Muller’s ratchet

## Abstract

The ultimate measure of viral fitness is the ability to maintain high prevalence within its host species. Effective transmission, efficient replication, and rapid immune evasion all contribute to this outcome. Over the past five years, severe acute respiratory syndrome coronavirus 2 has successfully adapted to humans, establishing long-term reservoirs and enabling sustained coexistence with the human population. We have observed innovative, synergistic mutations in the spike (S) protein that enhance receptor binding. Adaptation to the upper respiratory tract has shortened the incubation period, thereby facilitating viral spread. These improvements have also enabled immune escape mutations, even when such changes compromise replicative fitness. Adaptive mutations have driven intermittent selective sweeps by dominant variants. However, there are limits to functional enhancement. The receptor binding affinity of the S protein appears to have peaked between 2022 and 2023. The accumulation of fixed mutations plateaued following the emergence of BA.2.86/JN.1 around late 2023 and early 2024. Purifying selection has been the dominant evolutionary force acting on nonsynonymous mutations in the Omicron lineage, and the overall fitness impact of missense mutations in key viral proteins has declined. Additionally, due to weak selection pressure on synonymous mutations, the codon adaptation index in humans has been decreasing among Omicron subvariants. As a result, Omicron lineages have replicated less efficiently in cell cultures compared to the original virus, and recent variants show further attenuation in animal models. In the human population, this attenuation is reflected in declining COVID-19-related mortality, despite persistently high infection rates.

## Introduction

**Figure 1.**
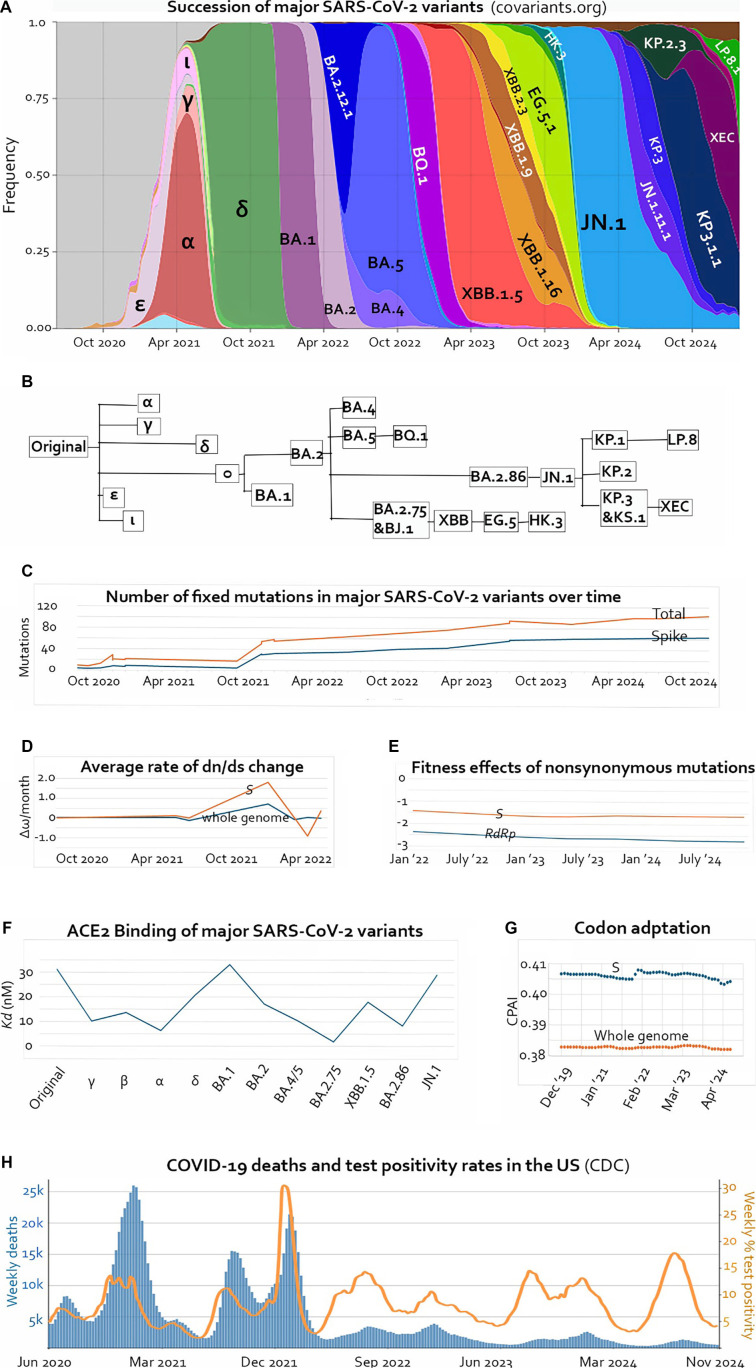
**Evolutionary timeline of major SARS-CoV-2 variants.** (A) Succession of dominant SARS-CoV-2 variants and dominant Omicron subvariants. Source: GISAID, via CoVariants.org (2025); (B) Phylogenetic relationship of lineages labeled in A; (C) Accumulation of fixed mutations in SARS-CoV-2 over time. Red: All proteins; Blue: The S glycoprotein. Mutation counts were obtained from outbreak.info [10]; (D) Average rate of dn/ds change from September 2020 to May 2022. Red: The *S* gene; Blue: Whole genome. Replotted with data from [8]; (E) Temporal change of fitness effects of nonsynonymous mutations from January 2022 to November 2024. Red: The *S* gene; Blue: The *RdRp* gene. Plotted with data from Bloom and Neher [6]; (F) Dissociation constants (*Kd*) of the S protein of major SARS-CoV-2 variants. Lower *Kd* values indicate higher receptor-binding affinity. Data obtained from [19] and [20]; (G) Change in CPAI of SARS-CoV-2 between December 2019 and July 2024. Blue: Coding sequence of the *S* gene; Red: All coding sequences. Plotted with data from [46]; (H) Weekly COVID-19 deaths and nucleic acid amplification test percent positivity in the United States from June 2020 to November 2024. Blue: Deaths; Orange: Test positivity. Data obtained from the Center for Disease Control and Prevention [56]. SARS-CoV-2: Severe acute respiratory syndrome coronavirus 2; CPAI: Codon pair adaptation index.

In the study of real-time evolutionary processes, no organism has been observed and documented as extensively as severe acute respiratory syndrome coronavirus 2 (SARS-CoV-2), the causative agent of COVID-19. Millions of genomic sequences have been analyzed throughout the pandemic. Due to its short generation time, high mutation rate, large surplus population, and strong natural selection, variants of SARS-CoV-2 have exhibited “speciation,” selective sweeps, and “extinction” within weeks and months. The remarkable evolution of different “dynasties” (Figure 1A and 1B) has provided valuable insights into the evolutionary trajectory of this zoonotic RNA virus and its interaction with the human host. It has also offered an opportunity to learn general trends in molecular evolution that would typically take extensive geological time to become evident in higher organisms.

SARS-CoV-2 is the third zoonotic coronavirus of the century. While severe acute respiratory syndrome coronavirus 1 (SARS-CoV-1) has gone extinct and Middle East respiratory syndrome coronavirus (MERS-CoV) is reported sporadically in Saudi Arabia, SARS-CoV-2 has persisted globally, with infections remaining prevalent year-round. This virus has demonstrated that the ultimate measure of viral fitness is the capacity to maintain high prevalence rates in its host species. Effective human-to-human transmission, efficient replication within human cells, and rapid immune evasion are critical mechanisms contributing to this fitness. At the molecular level, innovative mutations have enhanced the S protein of SARS-CoV-2, increasing its binding affinity to its primary receptor on human cells, angiotensin-converting enzyme 2 (ACE2). This gain in molecular function may have led to a greater ability to infect cells in the upper airway rather than the lungs, as ACE2 is more concentrated in the nasal epithelium [1]. This tropism toward nasal tissues has facilitated viral shedding and likely contributed to a shortened incubation period and increased transmissibility. Furthermore, the S protein serves as the primary target for neutralizing antibodies, which is why it, particularly its receptor-binding domain (RBD), has experienced a higher rate of adaptive mutations compared to other viral proteins.

Nonetheless, functional improvements have both theoretical and practical limits. Early in the pandemic, Zahradník and colleagues demonstrated through *in vitro* evolution that there exists an optimal configuration of the S protein (RBD-62) with an ACE2-binding affinity that is 1000-fold greater than that of the wild type [2]. Many variants of SARS-CoV-2 have utilized mutations predicted by this *in vitro* experiment. Concurrently, the virus must continually modify the S protein to evade neutralizing antibodies, irrespective of the mutations’ effects on receptor binding. Additionally, the evolution of the viral genome is not solely driven by functional advantages but also influenced by the preferred directions of host-initiated mutagenic mechanisms, including RNA editing proteins such as Apolipoprotein B mRNA Editing Catalytic Polypeptide-like (APOBEC), Adenosine Deaminase Acting on RNA (ADAR), and zinc finger antiviral proteins (ZAP) [3–5]. A significant proportion of mutations are not subject to selection and are thus prone to random drift [6]. Consequently, ideal conformations, such as RBD-62, are likely unattainable in nature.

This review aims to address critical questions: Where does SARS-CoV-2 stand in its evolutionary trajectory after five years of circulation among humans? Should we anticipate the emergence of more transmissible or virulent variants in the future, or is the virus irreversibly losing replicative fitness and virulence? Here, we discuss several observations suggesting that the genomic evolution of SARS-CoV-2 has reached a plateau and may be on the decline.

## Accumulation of fixed mutations is slowing down

While the majority (over 60%) of the ∼30,000 nucleotides of the SARS-CoV-2 genome have mutated at least once [4, 7], only a small proportion of the mutations have been selected and fixed [8]. Most synonymous mutations are near neutral, while most nonsynonymous mutations are deleterious [6]. Most of the fixed mutations are in the S protein. By the beginning of 2025, SARS-CoV-2 had accumulated over 60 mutations in the 1273-residue-long S protein, 30 of which are in the 223-residue-long RBD [9, 10]. On the other hand, there are altogether about 100 fixed nonsynonymous mutations in the SARS-CoV-2 genome coding for 29 proteins totaling over 9000 amino acid residues [10]. In other words, the overall rate of fixed amino acid mutations is ∼1%, while there are ∼5% fixed mutations in the S protein and over 13% in the RBD.

### Cooperative emergence of multiple affinity-enhancing mutations followed by immune escape mutations

Buildup of missense mutations in the *S* gene has been gradual, with two brief periods of acceleration: one at the emergence of Omicron BA.1 in late 2021 and the other at the emergence of Omicron BA.2.86 in 2023 (Figure 1C) [10]. The sudden increase in missense mutations in BA.1 also manifested as an increase in the ratio between nonsynonymous mutations and synonymous mutations (dn/ds or ω, Figure 1D).

Although many missense mutations in the S protein experienced positive selection [8], only about a dozen of them enhanced receptor binding affinity [11–15]. Synergism and epistasis among affinity-enhancing mutations played a major role in the mutation count jumps, meaning certain mutations facilitated the fixation of other mutations, generating new modes of interactions between the RBD and ACE2 [2, 16, 17]. The mutation burst in Omicron in 2021 provides a dramatic example of such epistatic interactions. Most notably, the N501Y mutation turned the affinity-reducing Q498R mutation into an affinity-enhancing mutation. Synergism between N501Y and Q498R reconfigured the RBD-ACE2 interaction in the Omicron variant [18]. Synergistic affinity-enhancing mutations are often followed by immune escape mutations, which provide more prominent selective advantages than affinity enhancement [12, 19–21]. The affinity-enhancing mutations in BA.1 (S477N, Q493R, Q498R, N501Y) first appeared in the original Omicron variant (B.1.1.529), and the affinity-reducing mutations (including three deletions) were added subsequently for immune escape, enabling BA.1 to spread quickly, displacing the Delta variant in a few months (Figure 1A) [10, 12].

The second, less dramatic, epistatic mutation burst occurred in 2023. The spike mutation R403K did not increase ACE2-binding affinity in the earlier B.1.1 lineage but did in the BA.2 lineage background [22]. It first appeared in BA.2.86. Although BA.2.86, with its high receptor-binding affinity, was recognized for its increase in frequency, it never rose to dominant status (with a peak frequency of ∼9% in the U.S. [23]). However, one more mutation, L455S, turned BA.2.86 into JN.1, which quickly wiped out all other subvariants to dominate the variant landscape (Figure 1A and 1B). L455S significantly reduced the ACE2-binding affinity of the RBD but provided JN.1 with strong immune-evading ability, along with enhanced fusogenicity and improved cell entry [20, 21, 24].

Epistatic mutations continued in the JN.1 sublineages but did not result in comparable mutation leaps or selective sweeps. Q493E enhanced ACE2 binding only in the presence of L455S and F456L, found in some JN.1 sublineages. Q493E emerged independently in KP.3 and LP.8 [17]. Subsequently, a deletion, S31del, developed in KP.3.1 to produce KP.3.1.1. Although the deletion itself reduced the ability of the virus to infect cells by membrane fusion, it granted KP.3.1.1 a significant competitive edge over its parent by immune evasion [25, 26]. S31del also independently emerged in other JN.1 sublineages such as KP.2.3 and LP.8, showing that S31 was a prominent immune target in the human population during 2024.

The historical pattern of SARS-CoV-2 evolution seems to indicate that there was more room for revolutionary innovations through synergistic mutations during the pioneering stages of a zoonotic epidemic. Later attempts at epistatic changes ended up only reformative.

### Convergence, flip, and reversion

Even though the S protein experienced strong positive selection [8], natural mutations fixed during the pandemic indicated that the virus has limited modes of receptor binding to which it can evolve. As a result, mutations in the *S* gene tend to be convergent, recurrent, and cyclical. Table 1 lists some of these recurrent S mutations, demonstrating that multiple variants resorted to the same strategies repeatedly.

**Table 1:** S mutations fixed in multiple independent lineages

**Mutations**	**Pre-alpha**	**Alpha**	**Beta**	**Gamma**	**Delta**	**Lambda**	**Omicron**
HV69-70del	B.1.258, B.1.375	x					BA.1
Y144del		x					XBB.1.5
R346							BQ.1.1, XBB.1.5
K417N/T			x	x			x
G446S							BA.1, BA.2.75
L452R/Q					x	x	BA.4/5
F456L							Multiple XBB and JN.1 sublineages
T478K					x		x
E484A/K			x	x			x
F490S						x	XBB.1.5
N501Y		x	x	x			x
D614G	A and B.1						
P681R/H		x			x		x

Fixation is defined as complete replacement in an entire population. Presence in all isolates of a variant is indicated with “x”. If fixed only in some subvariants, names of selected subvariants are given.

The FLip mutants revealed that the virus can swap the positions of two adjacent amino acids in the S protein to evade neutralizing antibodies and enhance receptor binding. Multiple XBB and JN.1 sublineages altered the positions of L455 and F456 through the FLip mutations (L455F + F456L) [16, 27]. Both mutations, particularly L455F, reduced receptor binding when acting alone [16], and F456L also decreased viral infectivity [15], likely explaining why they did not emerge early in the pandemic. However, more than three years into the pandemic. F456L appeared, presumably because it allowed the virus to evade antibodies developed against earlier lineages. The subsequent L455F mutation effectively restored receptor binding affinity and further aided the virus in penetrating herd immunity [16].

RBD mutations also reverted depending on the genetic background. The Q493R mutation in BA.1 and BA.2 was found to enhance receptor binding [28]. However, this mutation reverted in BA.2.75 and its XBB sublineages, as well as in BA.2.86 and its JN.1 sublineages. This reversal reduced receptor-binding affinity [29] but was feasible given the overall strong binding of the BA.2.75 and BA.2.86 lineages for immune evasion. In addition to Q493, six other amino acids in the RBD are known to experience “Yo-Yo” mutations. Notably, G446 and N501 mutated between two forms three times, while L452 mutated four times by August 2023. Meanwhile, the deletion of HV69-70 in the N-terminal domain of the S protein appeared and disappeared three times within the same timeframe [30].

### Natural restrictions on the improvement of receptor-binding affinity

Although *in vitro* evolution produced an RBD with a 1000-fold increase in receptor-binding affinity, the highest affinity we have observed in natural variants was no more than 20-fold stronger than wild type. The strongest receptor-binding virus in various reports was BA.2.75, XBB.1.5, or BA.2.86, depending on measurement methods and samples analyzed [14, 19–21, 24, 31]. Figure 1F shows dissociation constants (*Kd*) of the major variants according to [19] and [20], with lower *Kd* indicating higher binding affinity. The discrepancy between *in vitro* evolution and the actual *in vivo* outcomes attests to the differences between isolated molecular interactions on the surface of yeast cells and the natural selection of replicating viruses with competing functional priorities in the host cell and the need to compromise under immune pressure. For example, the D614G mutation, which became dominant globally within the first few months of the pandemic [32], resulted in an RBD that bound less tightly to ACE2 than wild type, but the mutation increased the density of intact S trimers on the viral surface by preventing premature dissociation of S1 from S2 following furin cleavage in the producing cell [33, 34]. As discussed earlier, after high-affinity RBDs were produced, they were quickly deoptimized by immune escape mutations that gave the lineages bigger advantages in human populations. The current trend shows an overall tendency of decrease in receptor-binding affinity (Figure 1F) [20, 21, 25, 35].

Of the nine RBD mutations in the optimal *in vitro* evolution product, RBD-62 [2], five of them are found in the current LP.8.1* lineage. Interestingly, although V445K in RBD-62 has never been reported in nature, mutation of V445 to another basic amino acid, histidine, has been fixed in the BA.2.86/JN.1 lineage. Recently, H445 was replaced by the third basic amino acid, arginine, in LP.8 and enhanced its receptor-binding affinity [35]. Another mutation in RBD-62, I468F, was found in early variants but was not fixed because it rendered the virus more susceptible to antibody neutralization [36]. The other two mutations in RBD-62 (I358F and T470M) have never been reported in natural variants, suggesting that these residues must be conserved for effective viral replication or for infection of the human host. Two mutations found in another *in vitro* evolution product with improved ACE2 binding (RBD-71), R408D and K417V, have not been reported in nature, but R408S and K417N/T have been fixed in multiple lineages. The fact that the mutations are tolerated suggests that the chances of R408D and K417V emerging in the future exist, but they are unlikely because aspartic acid (D) is categorically different from serine (S), as is valine (V) from asparagine (N) and threonine (T). Nevertheless, it is worthwhile to analyze the impact of D408 and V417 in the context of the current JN.1 sublineages.

## Deleterious mutations lead to degeneration and attenuation

Even with enormous population sizes, exponential growth, and strong natural selection, viruses still experience significant genetic drift [37]. They are subject to Muller’s ratchet, which refers to the irreversible accumulation of deleterious mutations due to random genetic drift, particularly in asexual organisms, leading to reduced fitness [37, 38]. In addition to the high error rates of viral RNA-dependent RNA polymerases (RdRp) [39], mammalian cells employ mutagenesis as a defense mechanism to diminish viral fitness [3–5, 38]. All Variants of Concern (VOC) have derived directly from the ancestral virus rather than evolving from previous VOCs, indicating that these variants have lower adaptability compared to the ancestral strain. The ancestral virus and most early variants are no longer present in the human population, leaving the existing subvariants vulnerable to Muller’s ratchet, despite occasional recombination between circulating lineages.

### Declining mutational fitness effects

Bloom and Neher [6] estimated the fitness effects of SARS-CoV-2 mutations by comparing the independent occurrences of each mutation to an expected number based on mutation rates at the third nucleotide of four-fold degenerate codons. They found that the overall effects of nonsynonymous mutations in each gene were negative. Additionally, by plotting the calculated average fitness effects of nonsynonymous mutations in the *RdRp* and *S* genes over time, we demonstrate that the overall fitness effects of missense mutations declined during the Omicron variant’s prevalence, from early 2022 to the end of 2024 (Figure 1E). This decline aligns with the observation by Maiti and colleagues of a dn/ds ratio decrease within the Omicron variant of concern [8]. In other words, further mutations are increasingly detrimental to viral fitness rather than adaptive.

The large genome of SARS-CoV-2 includes regions where natural selection is relaxed. Bloom and Neher’s study also found that the fitness effects of nonsynonymous mutations in accessory proteins were comparable to those of synonymous mutations, indicating they are nearly neutral. This finding is consistent with the observed accumulation of missense and nonsense mutations in these genes [8, 40].

### Declining codon adaptation index (CAI) in the human host

Besides functional improvements and immune escape, another direction of viral evolution after entering a new host species is the optimization of codon usage within the host’s cells, which involves synonymous changes. Selections of nonsynonymous mutations typically take priority over codon usage optimization because the fitness effects of the latter are more subtle. Therefore, one might expect sacrifices in codon usage in genes where selective pressure for adaptive evolution is high. The CAI of SARS-CoV-2 in the human population initially declined in early variants, was restored in Omicron [41–45], and then decreased again [44].

After analyzing the CAI and the codon pair adaptation index (CPAI), Padhiar and colleagues reported no drastic net changes in the codon usage of SARS-CoV-2 genes from December 2019 to July 2024. However, when I plotted the temporal curves using their supplementary data, I found that the *S* gene experienced two phases of decline in both CAI and CPAI, with a sharp increase in between. Additionally, there was an overall negative correlation between CPAI and time (*P* ═ 0.0028, Figure 1G) [46]. This suggests that the evolution of the *S* gene must balance compromises between receptor-binding and immune evasion while also bearing the cost of deoptimized codon usage. The timing of the jump in CAI and CPAI for the *S* gene coincided with the emergence of Omicron. The large number of adaptive mutations and simultaneous optimization of codon usage in the original Omicron variant suggest a unique evolutionary mechanism that remains a puzzle today [38].

Using the same dataset, we also observed significant codon degeneration in the ORF1ab (which includes the *RdRp* gene) and the *N* gene, where multiple adaptive mutations have been documented [40, 47–49]. The CPAI of the whole genome also showed a decreasing trend, although this was not statistically significant (*P* ═ 0.15).

### Loss of proofreading function

The effects of mutations outside the S protein on viral evolution are generally less well understood. Some mutations have been fixed in current lineages. Mutations in transcriptional regulatory sequences (TRSs) may alter the number and quantity of subgenomic transcripts, most of which lead to truncations and deletions [40]. Among the extra-spike variations, mutations in non-structural protein 14 (NSP14) are the most pertinent to viral evolution and are relatively well studied.

NSP14 is a multifunctional protein. Its N-terminal exoribonuclease domain is responsible for proofreading during viral RNA replication. Mutations in this enzyme disrupt proofreading and accelerate mutation accumulation throughout the viral genome [50–52]. The impact of NSP14 mutations on viral mutation load is stronger than that of NSP7, NSP8, and NSP12, even though the latter proteins form the core RNA polymerase complex [51]. One NSP14 mutation, I42V, was fixed in BA.1 and all subsequent Omicron subvariants. A humanized mouse model revealed that I42V contributed to the attenuation of BA.1, along with other Omicron mutations in NSP5, NSP6, M, and E, sparing infected mice from lethal brain invasion [53]. The only mutations in BA.1 that increased virulence in the context of the wild-type virus were those in the N protein, but they did not affect virulence in the context of Omicron NSP14, M, and E. The attenuating effect of the NSP14 mutation highlights the destructive nature of uncontrolled mutagenesis.

Unlike the S protein, which, as a major immune target, experiences positive selection based on dominant antibodies in the human population, mutations in non-structural proteins are mostly neutral [8, 40, 52] and subject to random drift. Consequently, there is greater divergence in proteins such as NSP14 than is reflected by the number of fixed mutations. In the study by Hassan and colleagues [52], 962 nonsynonymous point mutations were found among the 1581 nucleotides of the *nsp14* gene, with only 110 of the 527 amino acids conserved. Yet, there has been only one fixed mutation in NSP14 after five years. Therefore, degeneration of non-structural genes may be more pronounced than what we observe in the lineage-defining mutation spectrums.

### Phenotypical attenuation

The accumulation of deleterious synonymous and nonsynonymous mutations led to the attenuation of SARS-CoV-2 as the pandemic progressed. There was a decline in replication efficiency in cell cultures from the early B.1.1 variant to Omicron [26, 54]. The Omicron subvariants, including some dominant XBB family members, BA.2.86, and JN.1, exhibited reduced pathogenicity in hamsters and mice compared to the ancestral B.1 variant [24]. Later Omicron subvariants were less virulent than their earlier counterparts, although BA.2.86 and JN.1 demonstrated more efficient replication in human nasal epithelial cells [22, 24].

The attenuation of SARS-CoV-2 in humans was reflected in decreased mortality rates over time. A rapid decline in the mortality rate was observed during the first few months of the pandemic [55]. COVID-19-related mortality in the United States has been consistently declining, despite high viral prevalence, as indicated by elevated test positivity rates (Figure 1H) [56]. This decrease in mortality does not correlate with vaccination efforts; intensive vaccination in 2021 failed to prevent peaks in deaths later that year, and the federal government ceased free vaccinations in September 2023 [57]. The mortality rate is partially associated with natural immunity. Following the Omicron wave in the winter of 2021 and 2022, most Americans became seropositive [58], and mortality has significantly decreased since then. However, the subsequent gradual decline from 2022 to the present is likely more related to viral attenuation than to herd immunity, due to declining vaccine coverage and the virus’s rapid immune escape.

A global meta-analysis indicated that the global case fatality rates for the ancestral virus, Alpha (B.1.1.7), Beta (B.1.351), Gamma (P.1), Delta (B.1.617.2), and Omicron VOCs were 3.64%, 2.62%, 4.19%, 3.60%, 2.01%, and 0.70%, respectively [59]. It is noteworthy that the more lethal Beta and Gamma variants emerged before Alpha, and Delta was not as virulent as initially reported in Asia [60]. The case fatality rates for the ancestral virus, as well as the Alpha, Delta, and Omicron VOCs in North America, were 4.77%, 2.67%, 2.50%, and 0.73%, respectively, which aligns roughly with Figure 1H, considering the lag time between infection and death and the varying prevalence rates of the variants [59]. Both viral attenuation and the buildup of immunity likely contributed to the decrease in case fatality rates from the original virus to Omicron.

### Effect of vaccination on viral evolution

Vaccination is unlikely to affect mutagenic mechanisms; however, enhanced immune responses in vaccinated individuals help suppress the wild type, giving mutants a selective advantage even if they have reduced replicative fitness. A retrospective study indicated that vaccination was associated with an increase in viral diversity in India during 2021 and 2022 [61]. Furthermore, viral lineage diversity, measured as Shannon entropy, was higher in patients with vaccine breakthrough infections than in unvaccinated patients. The higher incidence of intra-host single nucleotide variants (iSNVs) in breakthrough infections suggested that vaccination accelerated viral evolution in the human host. Immune escape mutations were fixed at higher frequencies in vaccinated patients, and higher dn/ds ratios of viral isolates from vaccinees indicated stronger positive selection. Notably, when iSNVs of various viral lineages were compared, the early variants B.1 and B.1.1 exhibited much higher diversity than the VOC in both vaccinated and unvaccinated hosts, indicating faster evolution during the early phases of the pandemic as the virus adapted to humans.

We should not be concerned that vaccination facilitates the selection of immune escape mutations, as mutations occur regardless. Just as we do not refrain from using molnupiravir as an antiviral treatment despite its mutagenic effects, we should not avoid vaccination, which ultimately reduces the virus’s replicative fitness by forcing it to mutate.

## Conclusion

Currently, in the sixth year of the COVID-19 pandemic, there are increasing signs that the adaptive evolution of SARS-CoV-2 has plateaued. This is evident in the slower accumulation of fixed mutations, recurrent and cyclic mutations at the same sites, declining mutational fitness effects, reduced receptor-binding affinity of the S protein, diminishing codon optimization in the human host, and decreasing mortality rates. Furthermore, the evolution of SARS-CoV-2 has been gradual since the JN.1 sweep in early 2024, with only one addition to WHO’s Variants under Monitoring (VUM), LP.8.1*, since September 2024. The last addition to the Variants of Interest (VOI) was JN.1 in December 2023, while the last addition to the VOC was Omicron (B.1.1.529) in November 2021. Currently, LP.8.1.1 is poised to slowly displace the other JN.1 sublineages and dominate in the coming months. Like its predecessors, the new subvariant is expected to experience genetic drift under immune pressure as well as Muller’s ratchet.

SARS-CoV-2 may eventually resemble the four current human coronaviruses that cause the common cold [62]. If the Russian flu of 1889–1894 was indeed caused by a coronavirus that evolved into today’s HCoV-OC43, the five-year timeframe of that historical pandemic serves as an interesting reference point for COVID-19. Despite the role of airplanes in facilitating the global spread of SARS-CoV-2 and vaccines aimed at “flattening the curve,” the natural courses of these two respiratory illness pandemics have turned out to be comparable.
